# Editorial Note: Risk of stillbirth in singleton fetuses with advancing gestational age at term: A 10-year experience of late third trimester prenatal screenings of 50,000 deliveries in a referral center in northern Italy

**DOI:** 10.1371/journal.pone.0339421

**Published:** 2026-01-05

**Authors:** The PLOS One Editors

The *PLOS One* Editors issue this Editorial Note because this article [1] was identified as one of a series of submissions for which we have concerns about aspects of the peer review process. Readers are advised to interpret the article [1] with caution.

The Editors have no evidence of any author involvement in the peer review concerns.
